# Analysis of the current situation and factors influencing the utilization of somatic and somatic-mental disorder comorbidity and health services among middle-aged and older adult people in China

**DOI:** 10.3389/fpubh.2025.1536205

**Published:** 2025-04-07

**Authors:** Mo Xue, Qianqian Zhang, Jialing Wu, Kan Tian

**Affiliations:** ^1^School of Health Economics and Management, Nanjing University of Chinese Medicine, Nanjing, China; ^2^School of Management, Lanzhou University, Lanzhou, China; ^3^School of Elderly Care Services and Management, Nanjing University of Chinese Medicine, Nanjing, China

**Keywords:** somatic chronic disease comorbidity, somatic-mental disorder comorbidity, health service utilization, influencing factors, middle-aged, older adult people

## Abstract

**Background:**

The aging process has led to a significant increase in the prevalence of somatic chronic diseases (e.g., cardiovascular diseases, diabetes) among the middle-aged and older adult population. Additionally, this demographic is also susceptible to mental disorders (e.g., depression, anxiety). However, most studies in China focus on somatic chronic disease comorbidities, with less attention on comorbidities between somatic and mental disorders, as well as health service utilization.

**Purpose:**

To investigate the factors influencing the comorbidities of somatic and somatic-mental disorder and the status of health service utilization among middle-aged and older adult individuals in China.

**Methods:**

Data from the 2020 China Health and Retirement Longitudinal Study (CHARLS) were analyzed, including 16,565 middle-aged and older adult individuals. Among them, 9,498 had somatic chronic disease comorbidities, and 4,577 had somatic-mental disorder comorbidities. Logistic regression was used to analyze factors influencing comorbidity and health service utilization. Spatial distribution maps were created using ArcGIS 10.8 software, and association rules were mined using IBM SPSS Modeler 18.0 and R 4.4.1.

**Results:**

The prevalence of somatic chronic disease comorbidity (57.34%) was higher than that of somatic-mental disorder comorbidity (27.63%). The patterns of comorbidity exhibited a complex network structure, with arthritis or rheumatism, hypertension, and stomach or other digestive disorders serving as core nodes. Dispositional factors (e.g., gender, age) and demand factors (e.g., number of comorbidities, self-rated health) had a significantly greater influence on the risk of comorbidities and health service utilization behaviors than enabling factors (e.g., household income, basic health insurance). Patients with somatic-mental disorder comorbidities had 1.09 times higher outpatient utilization compared to those with somatic chronic disease comorbidities (95% CI, 1.01 to 1.18). The average number of outpatient visits was also higher for the somatic-mental disorder comorbidities (2.55 ± 2.81 visits) than the somatic chronic disease comorbidities (2.34 ± 2.69 visits).

**Conclusion:**

Pay attention to the variations in the distribution of various combinations of comorbidity patterns within the population and develop targeted treatment strategies and preventive measures. Emphasize enhancing patients’ self-management skills and health literacy, also considering economic factors. Elevate the significance of mental health services and management, strengthen patients’ social support networks, and reduce social stigma and discrimination.

## 1 Introduction

In the context of the global demographic transition, China is experiencing an unprecedented aging process, which has profound implications for both the public health system and individual health outcomes. Aging leads to a significant increase in the older adult population. Concurrently, the incidence of chronic diseases also rises (1), particularly the growing prevalence of comorbidities (2–4). These comorbidities present a substantial challenge to China’s public health infrastructure (5, 6). According to the 2020 Report on Nutrition and Chronic Disease of Chinese Residents, the prevalence of chronic diseases in China has been steadily increasing, with middle-aged and older adult populations exhibiting the highest rates of chronic disease (7, 8). The prevalence of multiple chronic diseases is reported to be 30.4% (9). The World Health Organization (WHO) defines chronic disease comorbidity as the simultaneous presence of two or more chronic conditions (10). The complexity, management challenges, and socioeconomic impact of comorbidities far exceed those associated with single chronic diseases (1, 11), making this phenomenon a focal point of global public health research.

Despite significant advancements in chronic disease comorbidity research in recent years, studies specifically focusing on developing countries, particularly China in the context of its rapidly aging population, remain limited. The Chinese Expert Consensus on the Management of Comorbidities in the Older Adult (2023) underscored the absence of clear guidelines for managing comorbidities, as well as a lack of sufficient research and practical applications in this area (12). In addition to the high prevalence of traditional physical chronic diseases such as cardiovascular diseases, diabetes, and chronic respiratory diseases, China’s older adult population also faces considerable mental health challenges, including depression and anxiety disorders (13, 14). The co-occurrence of both somatic and mental disorders necessitates particular attention. Data from the Blue Book of Psychiatric Mental Health in China 2023 indicated that 19.05% of older adults in China exhibited mild depression, while 12.17% displayed moderate to severe depressive symptoms (15). The prevalence of depressive symptoms among middle-aged and older adult individuals ranged from 32 to 37% (16), with those suffering from comorbidities at a higher risk of developing depression compared to individuals without such conditions (17).

The co-occurrence of somatic and mental disorder comorbidities not only increases clinical complexity (18) but also results in poorer treatment adherence, reduced quality of life (19), and overutilization of healthcare resources (20). This phenomenon is characterized by intricate, bidirectional interactions between physical and mental illnesses, involving complex causative mechanisms and influencing factors (21). Additionally, the long-term nature of these conditions often leads to socioeconomic burdens, pushing patients further into poverty and significantly diminishing their quality of life (22, 23). However, China’s “14th Five-Year” National Health Plan primarily focused on managing the “three highs” (hypertension, hyperglycemia, and hyperlipidemia), while systematic management strategies for comorbidities involving somatic-mental disorders are notably lacking (24). Domestic research in this area is relatively sparse, with most studies concentrating on the comorbidities of chronic somatic diseases rather than the intersection of somatic and mental disorders. Most studies focus on the comorbidities of somatic chronic diseases, primarily in three areas: first, exploring the fundamental principles and relevant influencing factors of somatic chronic disease comorbidities (25–27); second, examining the impact of somatic chronic disease comorbidities on health outcomes and health service utilization (7, 28); and third, revealing the complex patterns of comorbidities, as well as the associations and combinations of different diseases (7, 29). However, these studies rarely address both physical and mental comorbidities and often lack systematic management strategies for somatic-mental disorder comorbidities. Research on comorbidities involving mental disorders is relatively scarce; more often, mental disorders are explored independently or in combination with a single condition (24). Currently, only Wang et al. (30) have investigated the development trends and influencing factors of somatic-mental disorder comorbidities, while research on the combination of comorbidity patterns and their impact on health service utilization remains underdeveloped. There is a notable absence of comparative analyses between somatic and somatic-mental disorder comorbidities.

This study, grounded in Anderson’s health service utilization model (31, 32), explores the multivariate factors influencing somatic chronic disease comorbidities, somatic-mental disorder comorbidities, and health service utilization among middle-aged and older adult populations in China. It also compares health service utilization between these comorbidity patterns, examining predisposing, demand, enabling factors, and healthcare accessibility. By enhancing understanding of somatic and somatic-mental disorder comorbidities, this study provides valuable data and theoretical insights for comorbidity management, improving medical resource utilization, accessibility, and patient health outcomes. It also lays the foundation for developing a precise, personalized chronic comorbidity management system. It supports the realization of the “Healthy China 2030” strategic goal, contributing to the promotion of healthy aging.

## 2 Materials and methods

### 2.1 Participants

The data were obtained from the 2020 China Health and Retirement Longitudinal Study (CHARLS), which included 28 provinces (comprising autonomous regions and municipalities directly under the central government), 150 counties, and 450 villages (or communities). The study focused on middle-aged and older adult individuals aged 45 years and older, excluding those with memory-related conditions (e.g., Alzheimer’s disease, Cerebral atrophy, Parkinson’s disease), incomplete data on chronic diseases, missing scores on the Center for Epidemiological Studies Depression Scale (CES-D-10), and absent information regarding health service utilization (e.g., hospitalization in the past year, outpatient visits in the past month, number of hospitalizations, and number of outpatient visits). Ultimately, 16,565 participants were included, all of whom had been hospitalized in the past year and had visited outpatient clinics in the past month. Among these participants, 9,498 had somatic chronic disease comorbidities, and 4,577 had comorbid somatic-mental disorder comorbidities.

### 2.2 Research variables

Somatic chronic disease comorbidity was defined using the 12 self-reported categories of somatic chronic diseases in the CHARLS database. These categories include diabetes or elevated blood sugar, chronic lung disease, heart disease, stroke, kidney disease, asthma, hypertension, dyslipidemia, malignant tumors, liver disease, stomach or other digestive disorders, and arthritis or rheumatism. Comorbidity was considered present if an individual had ≥2 somatic diseases and no mental disorders. Somatic-mental disorder comorbidity was defined as the presence of ≥1 somatic disease and ≥ 1 mental disorder in the same individual (33, 34). Mental disorders were assessed as indicative of a depressive state if the score on the Center for Epidemiological Studies Depression Scale (CES-D-10) was ≥10 (34), based on the individual’s self-reported emotional and psychiatric symptoms.

#### 2.2.1 Dependent variables

(1) In examining the factors that influence the development of somatic chronic and somatic-mental disorder comorbidity, the explanatory variables “somatic chronic comorbidity” and “somatic-mental disorder comorbidity” were utilized. These variables were coded as follows: 0 = no comorbidity, 1 = presence of somatic or somatic-mental disorder comorbidity. (2) To investigate the factors affecting health service utilization among individuals with somatic chronic diseases and somatic-mental disorder comorbidity, the health status and functioning section of the 2020 CHARLES questionnaire was employed. The explanatory variables included “outpatient visits in the past month,” “number of outpatient visits,” “hospitalization in the past year,” and “number of hospitalizations.”

#### 2.2.2 Independent variables

The explanatory variables in this study were categorized into three perspectives: propensity factors, enabling factors, and demand factors, in accordance with Anderson’s health service utilization model. (1) Propensity factors encompass gender, age, marriage, residence, and education. (2) Enabling factors consist of household income and basic health insurance. (3) Demand factors include self-rated health, life satisfaction, sleep duration, the number of chronic disease comorbidities, as well as smoking status and alcohol consumption.

The number of chronic disease comorbidities was treated as an independent variable when examining the impact of somatic and somatic-mental disorder comorbidities on health service utilization.

### 2.3 Research methodology

#### 2.3.1 Statistical methods

Counts were expressed as relative frequencies, and group comparisons were conducted using the *χ*^2^ test. Continuous variables that meet the normality test were presented as $\bar{x}\pm S$ and compared using analysis of variance (ANOVA). Binary logistic regression models were utilized to analyze the factors influencing the development of somatic and somatic-mental disorder comorbidities, as well as health service utilization. The *χ*^2^ test and negative binomial regression were employed to compare health service utilization across various comorbidity patterns, with a significance level set at *α* = 0.05.

#### 2.3.2 Geographic information data

ArcGIS 10.8 software was employed to map the spatial distribution of somatic chronic disease comorbidity and somatic-mental disorder comorbidity prevalence among middle-aged and older adult populations in China. During this process, the prevalence data for somatic chronic diseases and somatic-mental disorders in these populations were accurately matched with the corresponding GIS vector data (in Shapefile format) of administrative regions, facilitating spatial visualization of the data. The prevalence rates were represented through a color-coded map, with darker colors indicating higher prevalence rates in the respective regions.

#### 2.3.3 Association rules

Association rules are a method for mining frequent patterns and relationships between two or more variables in complex datasets (35). The Apriori algorithm is commonly employed for iterative rule construction in the form of X → Y, where itemset X represents the antecedent and itemset Y represents the consequent, or the resulting association (36, 37). This process is based on three key metrics: support (X → Y) = P(X ∪ Y), confidence (X → Y) = P(X | Y) = P(X ∩ Y) / P(X), and lift (X → Y) = P(Y | X) / P(Y) = P(X ∩ Y) / [P(X)P(Y)]. Support measures the frequency of co-occurrence of X and Y, confidence evaluates the probability that Y occurs given X, and lift quantifies the strength of the association between X and Y (38). A lift value greater than 1 indicates that the association from X to Y is directional.

## 3 Results

### 3.1 Descriptive statistics

This study included a total of 16,565 participants, of whom 9,498 (57.34%) had comorbid somatic chronic diseases, and 4,577 (27.63%) had comorbid somatic-mental disorder. In the cohort with somatic chronic disease comorbidity, 35.57% were aged 65–74 years, 54.77% were female, 81.64% were married, 59.47% had rural household registration, 67.54% were non-drinkers, 58.50% were non-smokers, 44.65% had less than elementary school education, 52.05% reported a sleep duration of 6–8 h, and 54.57% had a household income of ≤3,000 yuan. In the cohort with somatic-mental disorder comorbidity, 36.01% were aged 65–74 years, 63.64% were female, 80.88% were married, 32.81% had rural household registration, 70.87% were non-drinkers, 64.43% were non-smokers, 51.41% had less than elementary school education, 44.26% reported a sleep duration of 6–8 h, and 58.03% had a household income of ≤3,000 yuan (Table 1).

**Table 1:** Baseline characteristics.

Variable		Somatic chronic disease comorbidity *N* (%)	Somatic-mental disorder comorbidity *N* (%)
Demographical characteristics			
Gender	Male	4,296 (45.23)	1,664 (36.36)
	Female	5,202 (54.77)	2,913 (63.64)
Marry	Married	7,754 (81.64)	3,702 (80.88)
	Other	1744 (18.36)	875 (19.12)
Residence	Countryside	5,650 (59.49)	3,116 (68.08)
	Municipalities	3,848 (40.51)	1,461 (31.92)
Age, years	45 ≤ Age < 55	1,414 (14.89)	782 (17.09)
	55 ≤ Age < 65	3,121 (32.86)	1,571 (34.32)
	65 ≤ Age < 75	3,378 (35.57)	1,648 (36.01)
	Age ≥ 75	1,585 (16.69)	576 (12.58)
Self-rated health	Very poor/poor	3,084 (32.47)	2009 (43.89)
	General	4,391 (46.23)	2,171 (47.43)
	Better/very good	2023 (21.30)	397 (8.67)
Education	Below primary school	4,241 (44.65)	2,353 (51.41)
	Primary school	2,103 (22.14)	1,038 (22.68)
	Middle school	1993 (20.98)	837 (18.29)
	High school and above	1,161 (12.22)	349 (7.63)
Retirement status	Yes	1904 (20.05)	554 (12.10)
	No	7,594 (79.95)	4,023 (87.90)
Household income, yuan	≤3,000	5,183 (54.57)	2,656 (58.03)
	≤10,000	2,726 (28.70)	1,237 (27.03)
	>10,000	1,589 (16.73)	684 (14.94)
Type of social health insurance	Medical insurance for urban workers	1,323 (13.93)	694 (15.16)
	Medical insurance for urban and rural residents	859 (9.04)	395 (8.63)
	Medical insurance for urban residents	427 (4.50)	209 (4.57)
	New Rural Cooperative Medical Insurance	6,202 (65.30)	2,925 (63.91)
	Other	223 (2.35)	114 (2.49)

### 3.2 Analysis of factors influencing the comorbidity of somatic and somatic-mental disorder in middle-aged and older adult people in China

The chi-square test for those with somatic chronic diseases comorbidities showed statistically significant differences in factors such as gender, marriage, self-rated health, alcohol consumption, life satisfaction, sleep duration, age, education, retirement status, and household income (*p* < 0.05). A binary logistic regression analysis, using the presence of somatic chronic disease comorbidity as the dependent variable (coded as 0 = no somatic chronic disease comorbidity, 1 = somatic chronic disease comorbidity) and the aforementioned factors as independent variables, indicated that being unmarried, alcohol consumption, older age, poorer self-rated health, shorter sleep duration, retirement status, and household income ≤10,000yuan were significantly associated with somatic chronic disease comorbidity (*p* < 0.05).

The chi-square test for those with somatic-mental disorder comorbidities showed statistically significant differences in factors such as gender, marriage, self-rated health, alcohol consumption, smoking status, sleep duration, age, education, retirement status, family income, and type of social health insurance (*p* < 0.05). The results of the binary logistic regression analysis, which considered the presence of somatic-mental disorder comorbidity as the dependent variable (coded as 0 = no somatic-mental disorder comorbidity, 1 = somatic-mental disorder comorbidity) and the aforementioned factors as independent variables, indicated that being female, unmarried, older, experiencing lower life satisfaction, having shorter sleep duration, possessing a lower education level, being retired, and household income ≤10,000 yuan were all associated with somatic-mental disorder comorbidity (*p* < 0.05) (Tables 2, 3).

**Table 2:** Comparative analysis of somatic and somatic-mental disorder comorbidity among middle-aged and older adult people in China.

Variable		Somatic chronic disease comorbidity		chi-square	*p*-value	Somatic-mental disorder comorbidity		chi-square	*p*-value
		Yes	No			Yes	No		
Gender	Male	4,296	3,502	30.40	<0.001	1,664	6,134	291.69	<0.001
	Female	5,202	3,565			2,913	5,854		
Marry	Married	7,754	6,124	75.08	<0.001	3,702	10,176	39.04	<0.001
	Other	1744	943			875	1812		
Residence	Countryside	5,650	4,249	0.69	0.407	3,116	6,783	182.10	0.100
	Municipalities	3,848	2,818			1,461	5,205		
Self-rated health	Very poor/poor	3,084	669	1675.84	<0.001	2009	1744	2305.22	<0.001
	General	4,391	3,212			2,171	5,432		
	Better/very good	2023	3,186			397	4,812		
Alcohol consumption	Yes	3,083	2,848	108.37	<0.001	1,333	4,598	122.80	<0.001
	No	6,415	4,219			3,244	7,390		
Smoking status	Yes	3,942	2,990	1.08	0.298	1,628	5,304	102.44	<0.001
	No	5,556	4,077			2,949	6,684		
Life satisfaction	Dissatisfaction	298	137	126.90	<0.001	267	168	1087.77	<0.001
	Less satisfactory	842	386			777	451		
	General	4,650	3,361			2,440	5,571		
	Relatively satisfactory	2,467	2,181			960	3,688		
	Very satisfied	394	347			133	608		
Sleep duration	≤5 h	3,924	2019	287.46	<0.001	2,321	3,622	606.34	<0.001
	6-8 h	4,944	4,513			2026	7,431		
	≥9 h	630	535			230	935		
Age group, years	45 ≤ Age < 55	1,414	2,103	133.09	<0.001	782	2,735	122.14	<0.001
	55 ≤ Age < 65	3,121	2,483			1,571	4,033		
	65 ≤ Age < 75	3,378	1706			1,648	3,436		
	Age ≥ 75	1,585	775			576	1784		
Education	Below primary school	4,241	2,857	41.89	<0.001	2,353	4,745	292.85	<0.001
	Primary school	2,103	1,536			1,038	2,601		
	middle school	1993	1,686			837	2,842		
	High school and above	1,161	988			349	1800		
Retirement status	Yes	1904	1,025	85.51	<0.001	554	2,375	135.19	<0.001
	No	7,594	6,042			4,023	9,613		
Household income, yuan	≤3,000	5,183	4,165	33.53	<0.001	2,656	6,692	10.51	0.005
	≤ 10,000	2,726	1784			1,237	3,273		
	>10,000	1,589	1,118			684	2023		
Type of social health insurance	Medical insurance for urban workers	1,323	992	0.65	0.958	694	1,621	10.37	0.035
	Medical insurance for urban and rural residents	859	648			395	1,112		
	Medical insurance for urban residents	427	304			209	522		
	New Rural Cooperative Medical Insurance	6,202	4,623			2,925	7,900		
	Other	223	174			114	283		

**Table 3:** Logistic regression analysis of somatic and somatic-mental disorder comorbidity among middle-aged and older adult people in China.

Variable	Somatic chronic disease comorbidity	Somatic-mental disorder comorbidity
	OR (95% CI)	OR (95% CI)
Gender (Male as a reference)		
Female	1.02 (0.94, 1.11)	1.69^***^ (1.48, 1.92)
Marry (Married as a reference)		
Other	0.90^*^ (0.80, 1.00)	0.85^**^ (0.76, 0.95)
Residence (Municipalities as a reference)		
Countryside		1.35^***^ (1.23,1.47)
Alcohol consumption (No as a reference)		
Yes	0.89^**^ (0.82, 0.97)	0.94 (0.85, 1.03)
Age Group, years (45 ≤ Age < 55 as a reference)		
55 ≤ Age < 65	1.60^***^ (1.45, 1.77)	1.35^***^ (1.21, 1.52)
65 ≤ Age < 75	2.30^***^ (2.06, 2.56)	1.47^***^ (1.30, 1.66)
Age ≥ 75	2.08^***^ (1.80, 2.41)	1.20^*^ (1.02, 1.40)
Self-rated health (poor/very poor as a reference)		
General	0.35^***^ (0.29, 0.35)	0.45^***^ (0.41, 0.49)
Better/very good	0.11^***^(0.10, 0.13)	0.17^***^ (0.15, 0.19)
Life satisfaction (dissatisfaction as a reference)		
Less satisfactory	1.26 (0.97, 1.65)	1.51^***^ (1.18, 1.94)
General	1.11 (0.88, 1.40)	0.52^***^ (0.41, 0.65)
Relatively satisfactory	1.09 (0.85, 1.38)	0.32^***^ (0.25, 0.40)
Very satisfied	1.20 (0.90, 1.59)	0.30^***^ (0.22, 0.40)
Sleep duration (≤5 h as a reference)		
6-8 h	0.79^***^ (0.73,0.86)	0.57^***^ (0.52,0.62)
≥9 h	0.67^***^ (0.57,0.78)	0.49^***^ (0.41,0.58)
Education (Below primary school as a reference)		
Primary school	1.02 (0.89, 1.17)	1.47^***^ (1.25, 1.73)
Middle school	0.97 (0.85, 1.10)	1.57^***^ (1.34, 1.84)
High school and above	1.01 (0.89, 1.15)	1.26^**^ (1.07, 1.47)
Retirement status (No as a reference)		
Yes	1.51^***^ (1.35, 1.68)	0.79^***^ (0.69, 0.89)
Household income, yuan (≤3,000 as a reference)		
≤10,000	1.10^*^ (1.01, 1.20)	0.92 (0.84, 1.01)
>10,000	1.05 (0.95, 1.17)	0.87^*^ (0.77, 0.97)

*Stands for *p*-value less than 0.05, **Stands for *p*-value less than 0.01, ***Stands for *p*-value less than 0.001.

### 3.3 Spatial distribution of the prevalence of somatic and somatic-mental disorder comorbidity among middle-aged and older adult people in China

**Figure 1:**
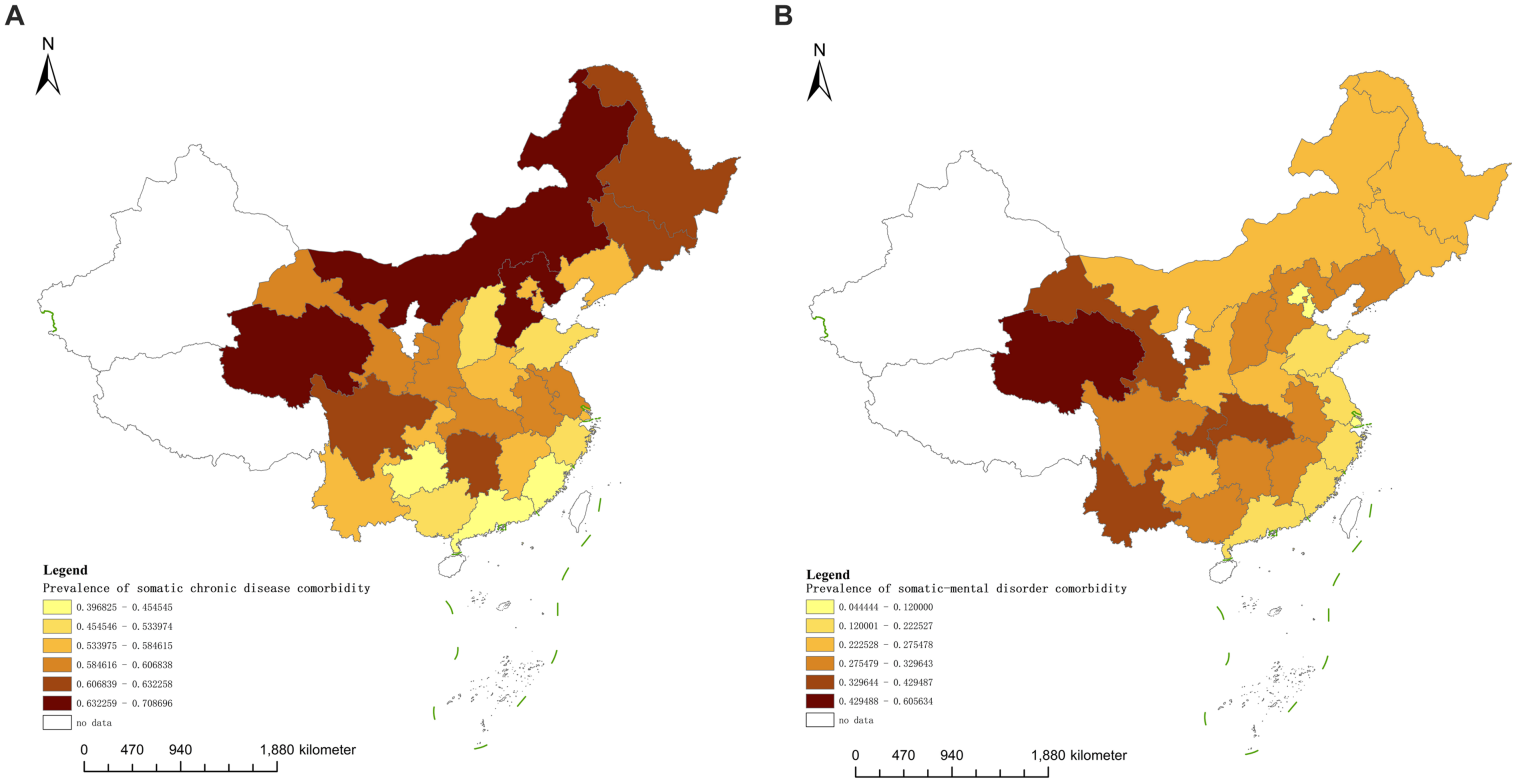
**(A,B)** Distribution of prevalence of somatic and somatic-mental disorder comorbidity among middle-aged and older adult people in China in 2020.

The prevalence of comorbidity of somatic chronic diseases among middle-aged and older adult individuals in China ranges from 39.68% (350/882 in Guangdong Province) to 70.87% (489/690 in the Inner Mongolia Autonomous Region). High-prevalence areas include the Inner Mongolia Autonomous Region, Qinghai Province, Hebei Province, Heilongjiang Province, and Hunan Province. In contrast, low-prevalence areas consist of Shanxi Province, Zhejiang Province, Guizhou Province, Fujian Province, and Guangdong Province. In most regions, prevalence rates fall between 50 and 70% (Figure 1A).

The prevalence of somatic-mental disorder comorbidity varies significantly, ranging from 4.44% (2/45 in Beijing) to 60.56% (86/142 in Qinghai Province). High-prevalence areas include Qinghai Province, Gansu Province, Hubei Province, Chongqing Municipality, and Yunnan Province. In contrast, low-prevalence areas encompass Fujian, Zhejiang, Shanghai, Beijing, and Tianjin. Most regions exhibit a prevalence rate between 20 and 40% (Figure 1B).

### 3.4 Analysis of comorbidity patterns of somatic and somatic-mental disorder among middle-aged and older adult people in China

**Figure 2:**
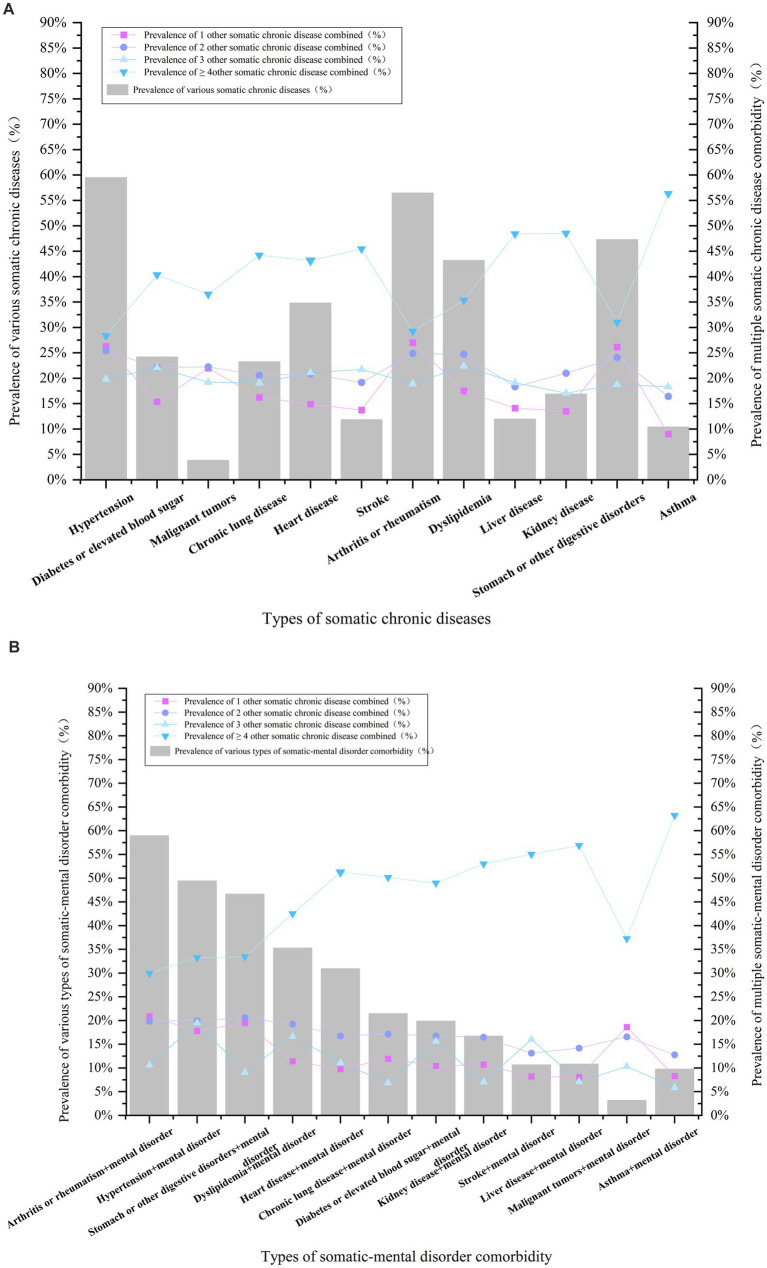
**(A,B)** Analysis of the pattern of comorbidity of somatic and somatic-mental disorder in middle-aged and older adult people in China.

The prevalence rates for the five most common somatic chronic diseases in the cohort were as follows: hypertension (5,649 cases, 59.48%), arthritis or rheumatism (5,361 cases, 56.44%), stomach or other digestive disorders (4,490 cases, 47.27%), dyslipidemia (4,100 cases, 43.17%), and heart diseases (3,304 cases, 34.79%). In terms of comorbidities associated with somatic chronic diseases, the highest number of combined comorbidities (≥4 other somatic chronic diseases) was observed in each category (Figure 2A).

**Figure 3:**
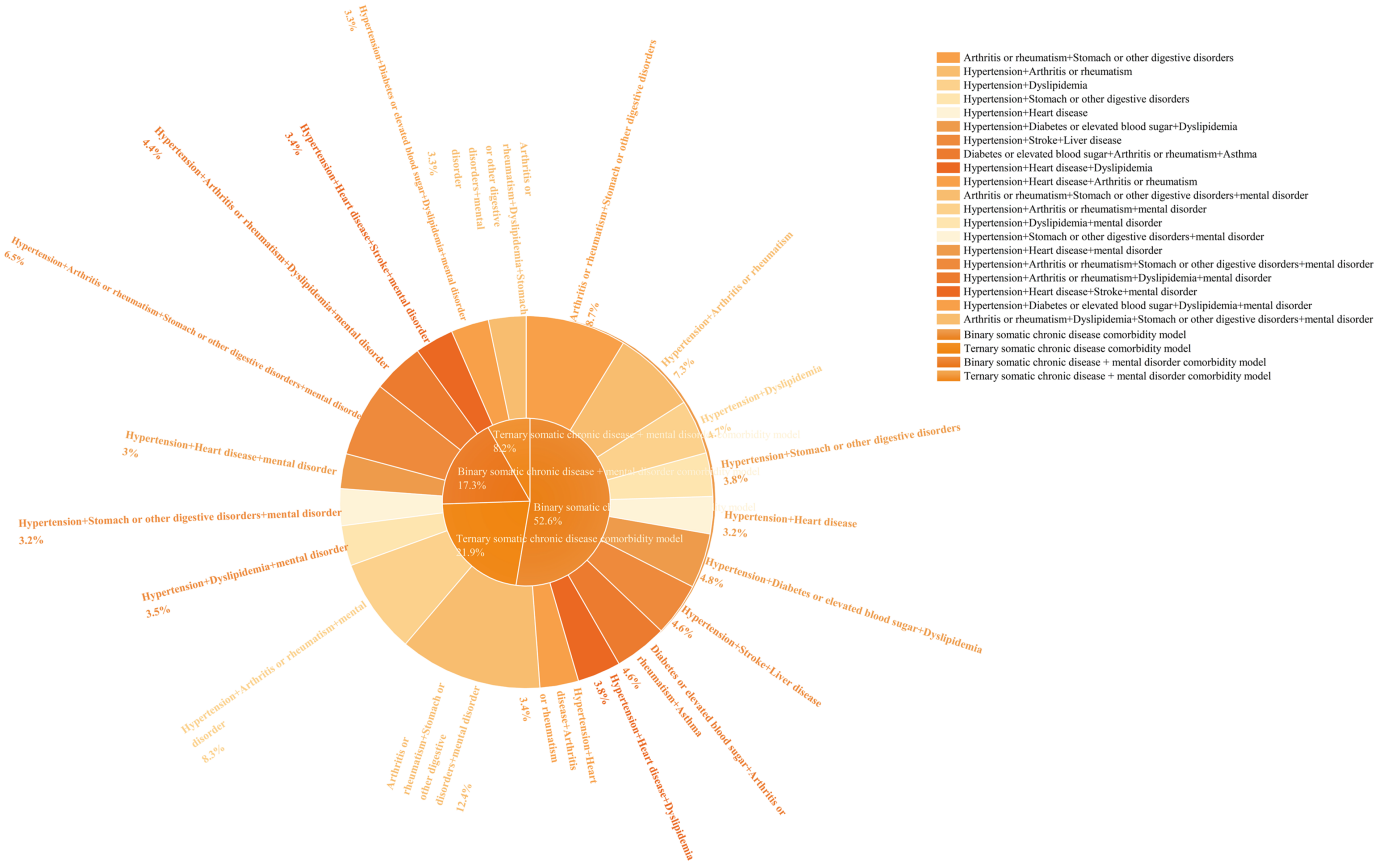
Percentage of combinations of somatic, somatic-mental disorder comorbidity patterns.

The analysis of comorbidity patterns reveals that the highest percentage of binary comorbidities occurred in the combination of arthritis or rheumatism with stomach or other digestive disorders, followed by hypertension in conjunction with arthritis or rheumatism. The most prevalent ternary comorbidities included hypertension, diabetes or elevated blood sugar, and dyslipidemia, followed by hypertension, stroke, and liver disorders (Figure 3).

Statistics on the co-occurrence of somatic-mental disorders indicated that the five most prevalent combinations are as follows: arthritis or rheumatism + mental disorder (2,697 cases, 58.93%), hypertension + mental disorder (2,261 cases, 49.40%), stomach or other digestive disorders + mental disorder (2,134 cases, 46.62%), dyslipidemia + mental disorder (1,614 cases, 35.26%), and heart disease + mental disorder (1,414 cases, 30.89%). The highest number of comorbid ≥4 other somatic chronic diseases was the highest in all categories (Figure 2B).

The pattern of somatic-mental disorder comorbidity was analyzed. In the binary comorbidity pattern, (arthritis or rheumatism + stomach or other digestive disorders + mental disorder) accounted for the highest percentage, followed by (hypertension + arthritis or rheumatism + mental disorder); and in the ternary comorbidity pattern, (hypertension + arthritis or rheumatism + stomach or other digestive disorders + mental disorder) accounted for the highest percentage, followed by (hypertension + arthritis or rheumatism + dyslipidemia + mental disorder) (Figure 3).

### 3.5 Analysis of comorbidity association rules for somatic and somatic-mental disorder in middle-aged and older adult people in China

Association rule analysis of 9,498 patients with somatic chronic disease comorbidities. The minimum conditional support was established at 3.0%, the minimum rule confidence was set at 50%, the minimum number of items was defined as 3, and the lift was required to be greater than 1. The association rules were ranked based on confidence, leading to the identification of the top five binary and tertiary somatic chronic disease comorbidity patterns.

The binary association rules were as follows: {asthma} → {chronic lung disease}, {stroke} → {hypertension}, {stomach or other digestive disorders} → {arthritis or rheumatism}, {arthritis or rheumatism} → {stomach or other digestive disorders}, and {diabetes or elevated blood sugar} → {dyslipidemia}. The ternary association rules were: {stroke, dyslipidemia} → {hypertension}, {diabetes or elevated blood sugar, heart disease} → {hypertension}, {diabetes or elevated blood sugar, dyslipidemia} → {hypertension}, {heart disease, dyslipidemia} → {hypertension}, and {asthma, stomach or other digestive disorders} → {chronic lung disease} (Table 4).

**Table 4:** Analysis of comorbidity association rules for somatic chronic disease in middle-aged and older adult people in China.

Antecedent (Math.)		Eventualities	Degree of support	Confidence level (Math.)	Promotion degree	Total number of cases
Binary somatic chronic disease comorbidity model	Asthma	Chronic lung disease	2.65	64.05	6.02	89
	Stroke	Hypertension	4.58	51.95	1.18	154
	Stomach or other digestive disorders	Arthritis or rheumatism	34.91	43.36	1.01	1,174
	Arthritis or rheumatism	Stomach or other digestive disorders	43.06	35.15	1.01	1,448
	Diabetes or elevated blood sugar	Dyslipidemia	10.50	23.80	1.12	353
Ternary somatic chronic disease comorbidity model	Stroke, Dyslipidemia	Hypertension	6.15	82.02	1.38	584
	Diabetes or elevated blood sugar, Heart disease	Hypertension	9.44	77.37	1.30	897
	Diabetes or elevated blood sugar, Dyslipidemia	Hypertension	15.19	75.81	1.28	1,443
	Heart disease, Dyslipidemia	Hypertension	17.26	74.50	1.25	1,639
	Asthma, Stomach or other digestive disorders	Chronic lung disease	5.20	74.49	3.21	494

Association rule analysis of 4,577 patients with somatic-mental disorder comorbidities. The minimum conditional support was established at 3.0%, the minimum rule confidence was set at 50%, the minimum number of items was defined as 3, and the lift was required to be greater than 1. The association rules were ranked by confidence level, resulting in the top five binary and ternary somatic-mental disorder comorbidity patterns.

The binary association rules were as follows: {asthma, mental disorder} → {chronic lung disease}, {stroke, mental disorder} → {hypertension}, {liver disease, mental disorder} → {arthritis or rheumatism}, {asthma, mental disorder} → {arthritis or rheumatism}, and {diabetes or elevated blood sugar, mental disorder} → {hypertension}. The ternary association rules were: {stroke, diabetes or elevated blood sugar, mental disorder} → {hypertension}, {stroke, dyslipidemia, mental disorder} → {hypertension}, {asthma, heart disease, mental disorder} → {chronic lung disease}, {asthma, stomach or other digestive disorders, mental disorder} → {arthritis or rheumatism}, and {diabetes or elevated blood sugar, heart disease, mental disorder} → {hypertension} (Table 5).

**Table 5:** Analysis of comorbidity association rules for somatic-mental disorder in middle-aged and older adult people in China.

Antecedent (Math.)		Eventualities	Degree of support	Confidence level (Math.)	Promotion degree	Total number of cases
Binary somatic chronic diseases + mental disorder model	Asthma, Mental disorder	Chronic lung disease	9.74	74.66	3.48	446
	Stroke, Mental disorder	Hypertension	10.64	73.31	1.48	487
	Liver diseases, Mental disorder	Arthritis or rheumatism	10.79	68.02	1.15	494
	Asthma, Mental disorder	Arthritis or rheumatism	9.74	67.71	1.15	446
	Diabetes or elevated blood sugar, Mental disorder	Hypertension	19.86	67.55	1.37	909
Ternary somatic chronic diseases + mental disorder model	Stroke, Diabetes or elevated blood sugar, Mental disorder	Hypertension	3.19	88.36	1.79	146
	Stroke, Dyslipidemia, Mental disorder	Hypertension	5.77	84.09	1.70	264
	Asthma, Heart disease, Mental disorder	Chronic lung disease	4.70	80.00	3.73	215
	Asthma, Stomach or other digestive disorders, Mental disorder	Arthritis or rheumatism	5.46	79.20	1.34	250
	Diabetes or elevated blood sugar, Heart disease, Mental disorder	Hypertension	8.65	78.79	1.60	396

### 3.6 Analysis of the current situation and factors affecting the utilization of health services for somatic and somatic-mental disorder comorbidity among middle-aged and older adult people in China

1. Among 9,498 middle-aged and older adult individuals with somatic chronic disease comorbidities, 2,490 (26.22%) had a one-year hospitalization, and 2,557 (26.92%) had one-month outpatient visits. The *χ*^2^ test showed that the number of somatic chronic diseases, marital status, self-rated health, alcohol consumption, life satisfaction, sleep, age, education, and family income had a statistically significant effect on hospitalization utilization (*p* < 0.05). Differences in the utilization of outpatient services based on the number of somatic chronic diseases, gender, self-rated health, alcohol consumption, life satisfaction, and sleep were also statistically significant (*p* < 0.05).

Logistic regression analysis was conducted based on Anderson’s health service utilization model, with hospitalization and outpatient visits as the dependent variables, and propensity factors, need factors, and enabling factors as independent variables. The logistic regression results for hospitalization showed that the likelihood of utilizing inpatient services was greater for those who had a higher number of comorbidities, were married, did not drink alcohol, were older, had lower life satisfaction, slept fewer hours, and had lower educational attainment. The logistic regression results for outpatient visits showed that the likelihood of utilizing outpatient services was greater for those who had a greater number of comorbidities, did not drink alcohol, had lower hours of sleep, and had lower life satisfaction (Table 6).

1. Among 4,577 middle-aged and older adult individuals with somatic-mental disorder comorbidities, 1,189 (25.98%) had a one-year hospitalization, and 1,312 (28.67%) had one-month outpatient visits. The *χ*^2^ test results showed that the number of somatic-mental disorder comorbidities, gender, age, marital status, self-rated health, alcohol consumption, smoking status, life satisfaction, sleep duration, exercise, and other variables had statistically significant effects on inpatient service utilization (*p* < 0.05). Differences in outpatient service utilization based on the number of somatic-mental disorder comorbidities, gender, self-rated health, alcohol consumption, smoking status, life satisfaction, and sleep duration were also statistically significant (*p* < 0.05).

**Table 6:** Analysis of factors influencing the utilization of health services for somatic and somatic-mental disorder among middle-aged and older adult people in China.

Variable	Somatic chronic disease comorbidity		Somatic-mental disorder comorbidity	
	Hospitalized or not	Outpatient or not	Hospitalized or not	Outpatient or not
	OR (95% CI)	OR (95% CI)	OR (95% CI)	OR (95% CI)
Number of comorbidities of chronic diseases (2 as a reference)				
3	1.23^**^ (1.61, 1.42)	1.31^***^ (1.14, 1.50)	1.39^**^ (1.09, 1.77)	1.28^**^ (1.03, 1.60)
4	1.60^***^ (1.37, 1.87)	1.60^***^ (1.37, 1.86)	1.72^***^ (1.35, 2.20)	1.82^***^ (1.45, 2.27)
5	2.06^***^ (1.73, 2.46)	1.85^***^ (1.56, 2.21)	2.08^***^ (1.61, 2.68)	1.90^***^ (1.50,2.41)
6	2.55^***^ (2.06, 3.16)	2.56^***^ (2.08, 3.16)	2.45^***^ (1.86, 3.23)	2.33^***^ (1.80, 3.03)
≥7	4.50^***^ (3.60, 5.63)	2.99^***^ (2.39, 3.72)	3.49^***^ (2.68, 4.54)	3.12^***^ (2.43, 4.00)
Gender (Male as a reference)				
Females		1.05 (0.91, 1.22)	1.42^**^ (1.14, 1.77)	1.00 (0.81, 1.23)
Marry (Married as a reference)				
Other	0.87 (0.77, 0.99)		0.97 (0.81, 1.16)	
Residence (Municipalities as a reference)				
Countryside				1.19^*^ (1.03, 1.37)
Alcohol consumption (No as a reference)				
Yes	0.78^***^ (0.70, 0.88)	0.88^*^ (0.78, 0.99)	0.70^***^ (0.59, 0.84)	0.97 (0.83, 1.14)
Smoking status (No as a reference)				
Yes		0.97 (0.84, 1.13)		0.84 (0.68,1.03)
Self-rated health (Poor/very poor as a reference)				
General	1.05 (0.94, 1.18)	1.70 (0.96, 1.20)	0.49^***^ (0.42, 0.57)	0.58^***^ (0.51, 0.68)
Better/very good	1.03 (0.87, 1.22)	1.03 (0.88, 1.22)	0.39^***^ (0.29, 0.54)	0.49^***^ (0.37, 0.66)
Age group, years (45 ≤ Age < 55 as a reference)				
55 ≤ Age < 65	1.24^*^ (1.05, 1.48)		1.24 (0.99, 1.56)	
65 ≤ Age < 75	1.68^***^ (1.42, 1.99)		1.54^***^ (1.23, 1.93)	
Age ≥ 75	2.20^***^ (1.80, 2.68)		1.99^***^ (1.51, 2.62)	
Life satisfaction (Dissatisfaction as a reference)				
Less satisfactory	0.73^*^ (0.54, 0.98)	0.97 (0.73, 1.30)		
General	0.62^***^ (0.47, 0.80)	0.76^*^ (0.59, 0.99)		
Relatively satisfactory	0.59^***^ (0.45, 0.77)	0.65^**^ (0.50, 0.85)		
Very satisfied	0.60^**^ (0.42, 0.85)	0.51^***^ (0.36, 0.73)		
Sleep duration, h (≤5 as a reference)				
6–8	0.81^***^ (0.73, 0.90)	0.83^***^ (0.74, 0.92)	0.96 (0.83, 1.12)	1.01 (0.88, 1.16)
≥9	0.85 (0.68, 1.06)	0.94 (0.75, 1.16)	0.85 (0.61, 1.18)	1.05 (0.76, 1.43)
Education (Below primary school as a reference)				
Primary school	0.82^*^ (0.69, 0.98)			
Middle school	0.90 (0.75, 1.08)			
High school and above	0.84 (0.70, 1.01)			
Exercise (No as a reference)				
Yes	0.81^*^ (0.68, 0.95)		0.84 (0.67, 1.04)	

*Stands for *p*-value less than 0.05, **Stands for *p*-value less than 0.01, ***Stands for *p*-value less than 0.001.

The logistic regression results for hospitalization showed that the greater the number of comorbidities, the female, the non-drinker, the poorer the self-rated health, and the older the population, the greater the likelihood of utilizing inpatient services. The logistic regression results for outpatient visits showed that as the number of comorbidities with chronic somatic-mental disorder increased, the likelihood of utilizing outpatient services also increased, particularly for individuals living in urban areas and those with poorer self-rated health (Table 6).

### 3.7 Analysis of the number of outpatient visits and hospitalizations for somatic and somatic-mental disorder comorbidities among middle-aged and older adult people in China

In the cohort of middle-aged and older adult individuals with somatic chronic disease comorbidities, 2,490 individuals had a history of hospitalization, with an average of 1.80 ± 2.73 hospitalizations. Regression models were applied to test the statistical significance of several variables, including the number of somatic chronic disease comorbidities, marital status, alcohol consumption, age, life satisfaction, sleep duration, and education. The results revealed that the number of somatic chronic disease comorbidities, age, and life satisfaction were significant factors influencing the number of hospitalizations, with statistically significant differences (*p* < 0.05).

A total of 2,557 middle-aged and older adult individuals had outpatient visits, with an average of 2.34 ± 2.69 visits. The regression analysis, which included variables such as the number of somatic chronic disease comorbidities, alcohol consumption, life satisfaction, and sleep duration, indicated that the number of somatic chronic disease comorbidities, alcohol consumption, life satisfaction, and sleep duration were significant factors influencing the number of outpatient visits, with statistically significant differences (*p* < 0.05) (Table 7).

**Table 7:** Basic analysis of the number of hospitalizations and outpatient visits for somatic chronic disease comorbidity among middle-aged and older adult people in China.

Variable		Number of hospitalizations ( $\bar{x}\pm s$ )	*F*-value	*p*-value	Number of outpatient visits ( $\bar{x}\pm s$ )	*F*-value	*p*-value
Number of comorbidities of somatic chronic diseases	2	1.60 ± 1.64	5.61	<0.001	2.30 ± 2.74	4.44	0.001
	3	1.73 ± 2.03			2.05 ± 2.11		
	4	1.67 ± 1.25			2.35 ± 2.65		
	5	2.13 ± 4.89			2.32 ± 2.46		
	6	1.89 ± 1.38			2.27 ± 1.72		
	≥7	2.21 ± 1.84			3.41 ± 4.42		
Alcohol consumption	Yes	1.74 ± 3.57	0.77	0.382	2.07 ± 2.04	10.35	0.001
	No	1.83 ± 1.76			1.74 ± 3.57		
Age group, years	45 ≤ Age < 55	1.49 ± 1.16	6.07	<0.001			
	55 ≤ Age < 65	1.84 ± 3.56					
	65 ≤ Age < 75	1.85 ± 1.97					
	Age ≥ 75	1.84 ± 1.31					
Life satisfaction	Very dissatisfied	2.19 ± 2.64	3.11	0.015	3.39 ± 3.64	3.73	0.005
	Less satisfactory	1.89 ± 1.25			2.70 ± 3.35		
	General	1.73 ± 1.79			2.26 ± 2.58		
	Relatively satisfactory	1.63 ± 1.46			2.21 ± 2.32		
	Very satisfied	1.63 ± 1.40			2.19 ± 1.75		
Sleep duration, h	≤5	1.85 ± 1.51	1.49	0.226	2.54 ± 2.87	6.33	0.002
	6–8	1.72 ± 2.92			2.16 ± 2.46		
	≥9	2.06 ± 3.09			2.28 ± 2.88		

In the cohort of middle-aged and older adult individuals with somatic-mental disorder, 1,189 individuals had a history of hospitalization, with an average of 1.80 ± 1.87 hospitalizations. Regression analysis was performed with variables such as the number of comorbidities with somatic-mental disorder, gender, self-rated health, alcohol consumption, and age. The results showed that the number of comorbidities with somatic-mental disorder and self-rated health were significant factors influencing the number of hospitalizations, with differences being statistically significant (*p* < 0.05).

1,312 middle-aged and older adult individuals had outpatient visits, with an average of 2.55 ± 2.81 visits. The regression analysis, which included variables such as the number of somatic-mental disorder comorbidities, self-rated health, alcohol consumption, and residence, revealed that the number of somatic-mental disorder comorbidities, alcohol consumption, and self-rated health were significant factors influencing the number of outpatient visits, with statistically significant differences (*p* < 0.05) (Table 8).

**Table 8:** Basic analysis of the number of inpatient and outpatient hospitalizations for somatic-mental disorder comorbidity among middle-aged and older adult people in China.

Variable		Number of hospitalizations ( $\bar{x}\pm s$ )	*F*-value	*p*-value	Number of outpatient visits ( $\bar{x}\pm s$ )	*F*-value	*p*-value
Number of comorbidities of somatic-mental disorder	2	1.55 ± 1.15	2.30	0.043	2.29 ± 2.92	2.79	0.017
	3	1.61 ± 1.50			2.61 ± 2.39		
	4	1.86 ± 2.72			2.30 ± 2.81		
	5	1.63 ± 1.00			2.69 ± 2.86		
	6	1.98 ± 2.36			2.38 ± 2.02		
	≥7	2.08 ± 1.69			2.91 ± 3.44		
Self-rated health	Very poor/poor	1.93 ± 1.75	4.42	0.012	2.98 ± 3.43	27.88	0.000
	General	1.59 ± 2.13			1.94 ± 1.39		
	Better/very good	1.62 ± 1.08			2.00 ± 1.56		
Alcohol consumption	Yes	1.70 ± 1.50	1.14	0.286	2.30 ± 2.08	5.03	0.025
	No	1.84 ± 1.96			2.63 ± 3.02		

### 3.8 Comparative analysis of indicators related to health service utilization for the two comorbidity types

A *χ*^2^ test was performed with inpatient/outpatient service utilization as the dependent variable and comorbidity pattern classification as the independent variable. The results showed that the difference in outpatient service utilization between the two comorbidity patterns was statistically significant (*p* < 0.05). Based on these results, a binary logistic regression analysis was conducted, revealing that the classification of the comorbidity pattern was a significant factor influencing outpatient service utilization. Specifically, outpatient service utilization was 1.09 times higher in the population with somatic-mental disorder comorbidities compared to the population with somatic chronic diseases (95% CI: 1.01 to 1.18).

A negative binomial regression was conducted with the number of hospitalizations/outpatient visits as the dependent variable and the comorbidity pattern classification as the independent variable. The analysis showed that the difference in the number of outpatient visits between the two patterns was statistically significant (*p* < 0.05), with the population with somatic-mental disorder having a higher number of outpatient visits compared to the population with somatic chronic disease comorbidities (Table 9).

**Table 9:** Comparison of health service utilization across comorbidity patterns.

Variable		Health service utilization			
		Hospitalization services [n (%)]	Outpatient services [n (%)]	Number of hospitalizations ( $\bar{x}\pm s$ )	Number of outpatient visits ( $\bar{x}\pm s$ )
Classification of comorbidity patterns	Somatic chronic disease comorbidity	2,493 (26.25)	2,557 (26.92)	1.80 ± 2.73	2.34 ± 2.69
	Somatic-mental disorder comorbidity	1,189 (25.98)	1,312 (28.67)	1.80 ± 1.87	2.55 ± 2.81
*X*^2^ (*F*) value		0.12	4.71	0.83	11.96
*p*-value		0.733	0.030	0.363	0.001

## 4 Discussion

### 4.1 Geographic variations and comorbidity patterns in China

The prevalence of somatic chronic disease comorbidities among middle-aged and older adult individuals in China was found to be as high as 57.34%, which is slightly lower than the 61.9% reported by Zhao et al. (39). In contrast, the prevalence of somatic-mental disorder comorbidities was 27.63%, a lower rate that has received less attention in China, despite existing studies indicating a comorbidity rate of approximately 30% (40, 41). These discrepancies may be attributed to variations in the exclusion criteria used for sample inclusion and the types of chronic diseases considered.

Geographically, both types of comorbidities exhibited higher prevalence rates in inland provinces, such as Inner Mongolia and Qinghai, while lower prevalence rates were observed in the coastal eastern provinces. This geographic distribution may be related to the level of economic development, the availability of medical resources, cultural and dietary habits, etc. Peters’ theory of Market Model emphasizes competition, and the role played by the private sector (42), but the model is not fully applicable to China’s healthcare sector, where most of the country’s healthcare institutions are mainly non-profit institutions, and emphasizes the dominant role played by the government’s financial inputs. Inland areas have a weak foundation for economic development compared to the eastern coastal provinces, resulting in less government financial investment in the health system, and imperfections in the health education of the population and the chronic disease prevention and control system. In addition, although the total amount of medical resources in China is steadily increasing, the geographical distribution is still not balanced, probably due to the complex terrain and inconvenient transportation in the inland areas, which limits the accessibility of medical resources to the residents. In addition to the above factors, dietary habits are also often considered an important factor affecting the prevalence of chronic diseases. Take Inner Mongolia, an inland province, for example, due to the influence of cultural factors, its drinking culture is prevalent, and to better adapt to the living environment, it is often characterized by a high-salt, high-fat diet, with a lack of vegetables and fruits, leading to a higher prevalence of related chronic diseases such as hypertension and dyslipidemia. The high prevalence of diseases in inland provinces is the result of the cycle of “low economic level - few medical resources - weak health awareness - heavy disease burden.” Although the overall prevalence of somatic-mental disorder comorbidity is relatively low, the areas with high prevalence remain concentrated in inland regions, underscoring the urgent need for enhanced mental health services in these locations. This situation also highlights the significant challenges these regions face in improving access to mental health care and optimizing the allocation of medical resources.

Both comorbidity patterns exhibit a complex network structure, with conditions such as arthritis or rheumatism, hypertension, and stomach or other digestive disorders serving as central nodes. This pattern aligns with findings from a study conducted on the middle-aged and older adult population in China, which identified prevalent multimorbidity patterns, including metabolic conditions (e.g., hypertension), gastrointestinal and hepatic disorders, as well as psychiatric and arthritic conditions (43). These diseases, characterized by high independent prevalence rates, are central to both binary and ternary comorbidity combinations. However, the Outline of the “Healthy China 2030” Plan focuses mainly on chronic diseases such as cancer, hypertension, and diabetes, and pays less attention to arthritis or rheumatism and stomach or other digestive disorders.

In the analysis of association rules, asthma, and chronic lung disease were identified as having strong correlations within the binary comorbidity pattern, which corresponds with the significant mortality risk these conditions present to Chinese adults (41). Bidirectional associations were noted for certain diseases, particularly metabolic disorders such as hypertension and diabetes or elevated blood sugar, with mental disorders complicating the relationships among somatic chronic diseases. A high prevalence of ≥4 co-existing somatic chronic diseases was observed in the comorbid population, reflecting the increasing complexity of multimorbidity. This not only emphasizes the severity of these patients’ health conditions but also highlights the urgent need for comprehensive management and intervention strategies.

China’s Medium-and Long-Term Plan for the Prevention and Treatment of Chronic Diseases (2017–2025) proposes exploring health management service models for chronic diseases and promoting the integration of the Internet and the health industry, so new medical service models such as telemedicine and mobile medical care can be promoted to break through geographic limitations, and in addition to the above measures, social forces can be encouraged to organize non-profit medical institutions in areas where medical resources are weak and there is a shortage of mental health care. In addition to the above measures, social forces can also be encouraged to organize non-profit medical institutions in areas with weak medical resources and a shortage of mental health, to optimize the pattern of medical resource allocation. In addition, in accordance with the “14th Five-Year Plan for National Health,” which points out the promotion of healthy lifestyles, the promotion of the “three reductions, three healths,” the implementation of reasonable diets, and other actions, health education can be carried out for the residents of inland areas to popularize the dangers of unhealthy dietary habits, and to encourage them to adopt a balanced nutritional dietary pattern. The Government should provide health education to inland residents, popularize the harm caused by unhealthy dietary habits, and encourage them to adopt a balanced nutritional diet. Secondly, the mental health service system should be strengthened, high-quality medical and healthcare personnel should be actively introduced and trained, and the level of medical services should be improved. Furthermore, it is important to consider the distribution characteristics of various comorbidity patterns and their evolving trends over time. Employing big data and epidemiological methods will help to identify high-risk groups, followed by comprehensive research into the intrinsic connections and pathogenesis of these diseases through multidisciplinary approaches, including genetics, molecular biology, and epidemiology. This approach will provide a robust foundation for developing targeted therapeutic strategies and preventive measures.

### 4.2 Propensity and demand factors significantly influence the risk of comorbidity and individual health service utilization behavior more than enabling factors

Propensity and need factors, including gender, alcohol consumption, age, education, self-rated health, sleep duration, and retirement status, significantly influence the development of somatic and somatic-mental disorder comorbidities. Regarding health service utilization, factors such as gender, place of residence, marital status, age, education, and the number of comorbidities, along with self-rated health and sleep duration, emerged as significant variables affecting individual health service utilization behavior. In contrast, enabling factors demonstrated a weaker impact on both the development of comorbidities and health service utilization. For instance, household income did not show a direct association with health service utilization, despite being identified as a potential influencing factor for both comorbidities.

In this study, it was found that women and individuals residing in rural areas exhibited a higher likelihood of developing somatic-mental disorder comorbidities. This finding aligns with both national and international research (44), indicating that biological, social, and cultural factors may contribute to this disparity (45). Furthermore, variations in personality traits may lead women to experience more negative emotions when confronted with adverse life events, thereby increasing their susceptibility to comorbidities (46). In rural areas, residents have a lower level of education, a lack of health education, and a relatively backward economy, resulting in imperfect medical facilities, a heavier burden of disease among residents, delayed or even no medical care, or medical care but not due to treatment, resulting in a “small disease to big, big disease to dangerous,” very likely to cause other complications, resulting in an increased risk of disease (47). In addition, they may face greater stress and challenges in life, and even some physical illnesses may have an impact on mental health (45), leading to an increased probability of psychological problems. In addition, Peters proposes the Participatory Model, which emphasizes citizen participation and is committed to seeking more democratic and collective mechanisms to send signals to the government (42). However, China’s 14th Five-Year National Health Plan places greater emphasis on improving service capacity in the field of mental health and promoting integrated management mechanisms, a top-down operational mechanism that lacks participation and feedback from citizens.

Furthermore, the utilization of health services was higher among individuals with a greater number of comorbidities, older age, non-alcohol consumption, shorter sleep duration, lower life satisfaction, and poorer self-rated health. These individuals often experience a diminished health-related quality of life, necessitating more prescriptions, medications, laboratory tests, and imaging, which in turn increases their demand for healthcare services (48). However, these factors do not operate in isolation, they are interconnected and collectively influence an individual’s health status and healthcare utilization behavior.

Although some enabling factors, such as household income, may not demonstrate a direct effect, they can still influence health service utilization by reducing financial barriers and enhancing patients’ willingness and ability to seek care (49). Therefore, when developing prevention, treatment, and rehabilitation strategies, a comprehensive, multidimensional approach should be adopted. The importance of propensity and demand factors should not be overlooked. The patient-centered principle should be adhered to, and a feedback and response mechanism should be established from the bottom to the top to address the actual needs of the residents, so that they can better participate in the construction of the mental health system, and secondly, focusing on improving the self-management ability of the patients and their level of health literacy, and reinforcing the construction of their social support system. Concurrently, economic factors, such as expanding basic medical insurance coverage and promoting family doctor systems, should be considered to achieve optimal treatment and rehabilitation outcomes.

### 4.3 Outpatient service utilization was significantly higher in the somatic-mental disorder comorbidity than in the somatic chronic disease comorbidity

Although the prevalence of somatic-mental disorder comorbidities was lower than that of somatic chronic disease comorbidities, the former group exhibited a trend of higher outpatient service utilization. Descriptive statistics and regression analysis indicated that patients with somatic chronic disease comorbidities utilized inpatient services more frequently, whereas those with somatic-mental disorder comorbidities relied more on outpatient services. Furthermore, a greater number of comorbidities, a more urban population, and poorer self-rated health were associated with increased utilization of outpatient services. This trend can be attributed to the distinct nature of the two types of comorbidities and their respective treatment modalities. Chronic somatic conditions often necessitate long-term treatment and monitoring, which is best provided through inpatient care, offering more comprehensive and systematic treatment options.

In contrast, somatic-mental disorder comorbidities are frequently linked to social stigma and discrimination (50), prompting patients to favor the privacy of outpatient services, where they can seek professional assistance without the psychological burden associated with hospitalization. Furthermore, the side effects of antipsychotic medications often worsen somatic conditions (51), making the treatment of psychiatric and physical health more interconnected, thereby increasing the demand for outpatient services.

Patients with co-occurring somatic-mental disorder also tend to have poorer self-management skills and lower health-related quality of life (52, 53). This population faces complex clinical management, not only due to the physical and mental burden of disease but also because of the need for interdisciplinary collaboration in treatment. Current models of specialized, mono-disease management fail to effectively minimize the impact of “disruptive medicine.” In international contexts, mental health disorders are integrated into the management of common diseases, as seen in countries like the U.S and the U.K (20, 54, 55). However, mental health services in China have not received sufficient attention. The “14th Five-Year Plan” still focuses primarily on the co-management of the “three highs” (hypertension, hyperglycemia, and hyperlipidemia), lacking systematic management strategies for somatic-mental disorder comorbidities (24).

Therefore, future policy development and practice should be based on the comprehensive prevention and control of chronic diseases proposed in the Medium-and Long-Term Plan for the Prevention and Control of Chronic Diseases in China (2017–2025), comorbidity management integrating somatic-mental disorder can be carried out with mental disorder as an entry point. Peters’ Flexible Government Model advocates that the government should be more adaptive and flexible to be able to adjust the corresponding policies promptly to face the challenges in a better way (42), so that, in future policy formulation, it is necessary to increase the importance of somatic-mental disorder comorbidity and emphasize the impact of bidirectional mechanisms of action between the two in terms of morbidity risk and disease burden. In addition, it is necessary to prioritize mental health services, establish a comprehensive and effective social support network for patients, reduce social stigma, and promote interdisciplinary collaboration. A patient-centered, comprehensive health management system encompassing prevention, diagnosis, treatment, rehabilitation, and psychological support is essential. Concurrently, scientific research should concentrate on the pathogenesis, risk assessment, and intervention strategies for somatic-mental disorder, thereby providing a solid foundation for more accurate and effective management strategies.

## 5 Conclusion

This study explored the prevalence, patterns, and factors affecting comorbidity risk and health service utilization among middle-aged and older adult individuals in China. Findings showed that somatic chronic disease comorbidity was significantly more prevalent than somatic-mental disorder comorbidity. Dispositional and demand factors influenced comorbidity risk and health service utilization more than enabling factors. These results underscore the challenge of managing multimorbidity in China’s aging population and highlight the need for an integrated healthcare approach addressing physical and mental health. Future research should focus on understanding somatic-mental disorder comorbidity and improving health service accessibility and quality. A patient-centered health management system that includes prevention, diagnosis, treatment, rehabilitation, and psychological support is crucial for addressing the complex health needs of this demographic.

## Data Availability

The original contributions presented in the study are included in the article/supplementary material, further inquiries can be directed to the corresponding author.
